# Supplementary figures and images for: Machine learning identifies genes linked to neurological disorders induced by equine encephalitis viruses, traumatic brain injuries, and organophosphorus nerve agents
Source: Front Comput Neurosci. 2025 May 13;19:1529902. doi: 10.3389/fncom.2025.1529902 (PMC12106541; doi:10.3389/fncom.2025.1529902)

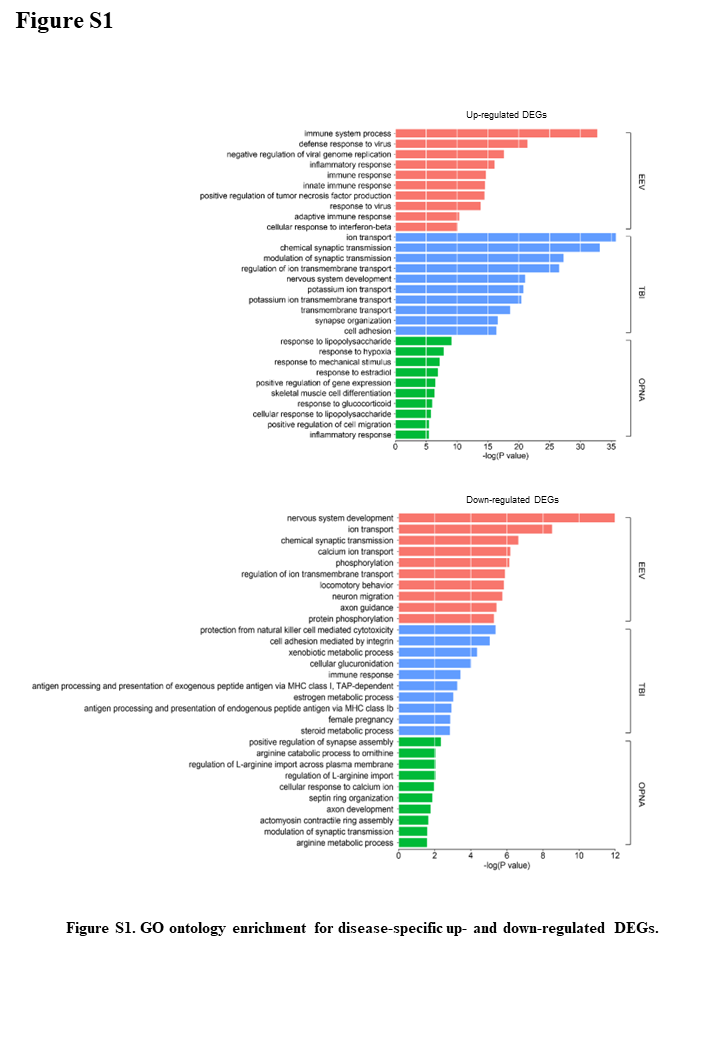

Supplement: Supplementary file 2 [file Image_1.png]
